# Approach for delabeling beta-lactam allergy in children

**DOI:** 10.3389/falgy.2023.1298335

**Published:** 2023-11-15

**Authors:** R. Sáenz de Santa María, G. Bogas, M. Labella, A. Ariza, M. Salas, I. Doña, M. J. Torres

**Affiliations:** ^1^ Allergy Unit, Hospital Regional Universitario de Málaga, Hospital Civil, Málaga, Spain; ^2^ Allergy Research Group, Instituto de Investigación Biomédica de Málaga-IBIMA, Hospital Civil, Málaga, Spain; ^3^ Nanostructures for Diagnosing and Treatment of Allergic Diseases Laboratory, Andalusian Center for Nanomedicine and Biotechnology-BIONAND, Parque Tecnológico de Andalucía, Málaga, Spain; ^4^ Departamento de Medicina, Universidad de Málaga, Facultad de Medicina, Málaga, Spain

**Keywords:** anaphylaxis, beta-lactam, children, drug provocation test, maculopapular exanthema, skin test, urticaria

## Abstract

A considerable number of pediatric patients treated with beta-lactam (BL) antibiotics develop delayed onset of skin rashes during the course of treatment. Although the most frequent cause of these symptoms is infectious, many cases are labeled as allergic reactions to these drugs. BL allergy labels could have a negative impact, as they imply avoidance of this group of drugs and the use of second-line antibiotics, leading to a potential increase in adverse effects and the utilization of less effective therapies. This constitutes a major public health concern and economic burden, as the use of broad-spectrum antibiotics can result in multidrug-resistant organisms and prolonged hospital stays. Therefore, it is crucial to delabel patients during childhood to avoid false labeling in adult life. Although the label of BL allergy is among the most frequent causes of allergy referral, its management remains controversial, and new diagnostic perspectives are changing the paradigm of managing BL allergies in children. Traditionally, drug provocation testing (DPT) was exclusively performed in patients who had previously obtained negative results from skin tests (STs). However, the sensitivity of STs is low, and the role of *in vitro* testing in the pediatric population is not well defined. Recent studies have demonstrated the safety of direct DPT without prior ST or serum tests for pediatric patients who report a low-risk reaction to BLs, which is cost-effective. However, there is still a debate on the optimal allergic workup to be performed in children with a benign immediate reaction and the management of children with severe cutaneous adverse drug reactions. In this review, we will discuss the impact of the label of BL allergy and the role of the different tools currently available to efficiently address BL allergy delabeling in children.

## 1. Impact of beta-lactam allergy as a public health problem

Up to 10% of children treated with beta-lactams (BLs) develop delayed maculopapular exanthema or urticaria (1). Although the most frequent etiology for these symptoms is infectious, with approximately two-thirds of children presenting with a confirmed viral illness (2, 3), most of the cases are labeled as penicillin allergies.

A penicillin allergy label directly impacts the selection of antibiotics, potentially leading to negative consequences such as a higher risk of antimicrobial treatment failure, developing antimicrobial resistance, occurrence of adverse drug reactions due to the use of a broader spectrum or alternative antibiotic, and increased healthcare costs (4–15). In this regard, it has been observed that approximately 50% of children who have been diagnosed with an antibiotic allergy are prescribed antibiotics that are not suitable for the specific infection being treated (16–19), placing patients at risk for the use of less effective therapies and an increased likelihood of treatment failures (20, 21). Moreover, the use of broader spectrum antibiotics can lead to increased rates of infection with multidrug-resistant organisms such as *Clostridium difficile* infection, vancomycin-resistant *Enterococcus*, and methicillin-resistant *Staphylococcus aureus* (4–6, 9, 13, 15, 16, 22–25). All of this leads to increased length of hospital stays compared with the general population (5–9). It has also been reported that children labeled as allergic to penicillin have a higher comorbidity index and incur higher hospitalization costs (23). Globally, this constitutes a substantial public health threat and economic burden (4, 5, 15, 16, 22–25).

Penicillin allergy labels are usually acquired during childhood, with up to 75% of patients labeled as allergic before the age of 3 years (26). This labeling often persists throughout adulthood. Indeed, up to 20% of the general population denominate themselves “penicillin-allergic” (26–29). However, less than 10% of them are confirmed as truly allergic after a proper allergy assessment (30–34). Therefore, there is the potential for a large majority of these allergies to be effectively “delabeled” (35, 36). Consequently, the evaluation and delabeling of BL allergy in pediatric population constitute important public health goals in order to avoid dragging that label into adult life, with the above-mentioned consequences. Moreover, the evaluation study of suspected penicillin allergic reactions in children is a cost-effective measure, as delabeling plays a critical role in promoting antimicrobial stewardship (37). In this regard, it has been estimated that subjects labeled as penicillin-allergic prior to age 10 years have lifetime antibiotic costs that are $2,000 higher compared with those who were not allergic to penicillin (38). In addition, the evaluation and subsequent removal of the penicillin allergy label in hospitalized patients prevented 504 inpatient days and 648 outpatient days on alternative antibiotics (39). Moreover, the removal of penicillin allergy label from 145 charts in an antimicrobial stewardship program in a tertiary care hospital resulted in an annual savings of $82,000 (40).

Despite the importance of delabeling to reduce adverse healthcare outcomes and decrease healthcare costs, barriers in tackling incorrect penicillin allergy labels have been identified, being among the most relevant barriers is the lack of knowledge of local pathways for evaluating antibiotic allergy (41). Therefore, there is a great need to provide antibiotic allergy education to non-allergy specialists, as well as to determine the best strategies to safely delabel children (42).

In this manuscript, we review the role of the different tools currently available to efficiently tackle BL allergy labels, including both *in vivo* [skin tests (STs) and drug provocation test (DPT)] and *in vitro* tests. The data sources utilized in this study consisted of English language literature obtained from MEDLINE, specifically focused on beta-lactam drug hypersensitivity in children. The selection of the studies was based on relevance, date of publication, and originality.

## 2. Immunochemistry and mechanisms involved in BL allergy

**Figure 1:**
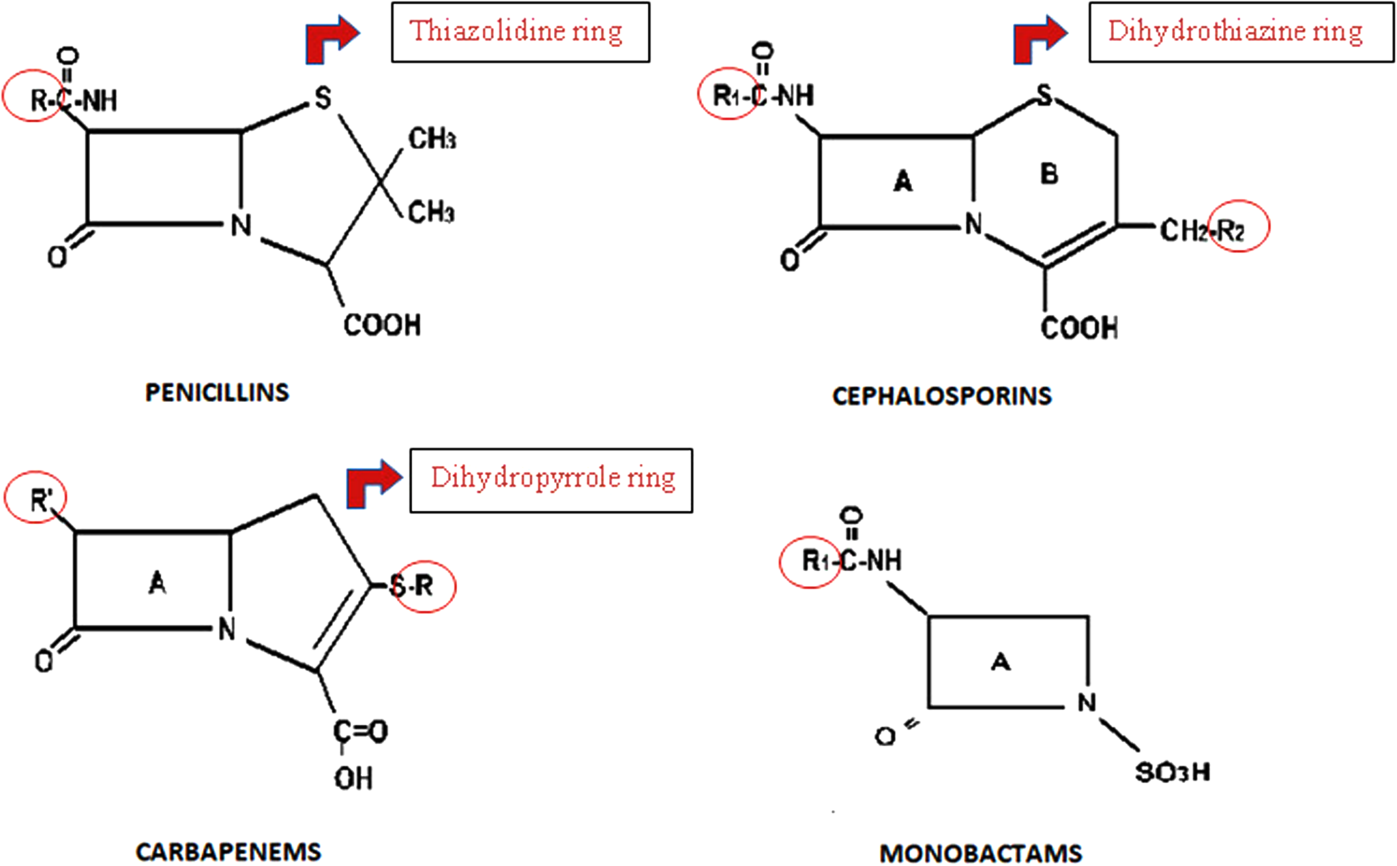
General chemical structure of BLs.

BL antibiotics are classified into five families: penicillins, cephalosporins, carbapenems, monobactams, and beta-lactamase inhibitors. All BLs share a common four-carbon ring called BL ring, but differ in the adjacent ring (a thiazolidine ring in penicillins, dihydrothiazine in cephalosporins, dihydropyrrole in carbapenems, and oxazolidine in beta-lactamase inhibitors). All of them, with the exception of beta-lactamase inhibitors, have an R1 side chain, which determines their antibacterial and pharmacokinetic action and is shared by some penicillins and cephalosporins. Cephalosporins and carbapenems also have a second R2 side chain. Monobactams have a monocyclic core, the only representative of which is aztreonam, which distinguishes it from other BLs (43) (Figure 1).

Hypersensitivity reactions can lead to any of the four immunologic effector mechanisms described by Coombs and Gell (44). Following penicillin administration, spontaneous opening of the beta-lactam ring occurs, giving rise to the different metabolites with the capacity to stimulate the immune system (43). They are low weight molecules that need to be conjugated to a carrier protein to induce an immune response (43, 45). Other immune activation mechanisms have also been described, wherein certain drugs are capable of binding to T cell or HLA receptors even in the absence of a hapten (46).

Clinically, the classification of hypersensitivity reactions to BL relies on the symptoms manifested in the reaction and their timing. In this sense, immediate reactions (IRs) occur within 1–6 h following administering the drug, while non-immediate reactions (NIRs) require a longer interval, usually after several hours or even days (47–49). IRs are usually IgE-mediated and manifest as urticaria, angioedema, rhinitis, bronchospasm, anaphylaxis, or acute gastrointestinal symptoms with abdominal pain, vomiting, and diarrhea. NIRs are related to a cellular mechanism and usually manifest as delayed urticaria, maculopapular rash, fixed drug rash, vasculitis, toxic epidermal necrolysis, Stevens–Johnson syndrome, drug reaction with eosinophilia and systemic symptoms, acute generalized rash pustulosis, symmetrical flexural intertriginous rash, or organ-specific involvement. Although this classification is not strict and overlaps exist, it proves to be valuable when considering the clinical evaluation and the diagnostic workup (43).

Allergic reactions may involve the BL ring, other rings, side chains, or allergenic determinants, which determines the cross-reactivity profile (50–53).

## 3. The value of clinical history

The first step in the approach of delabeling BL allergy in children involves a complete medical history, including questions regarding the patient, such as age or family history, as well as details regarding the reaction, symptoms, time of appearance, time of resolution, or drug implicated (54–57).

Concerning issues related to the patient, some studies have demonstrated that the age at the moment of the reaction is important. In this regard, confirmed allergic reactions to BLs is less frequent in children younger than 5 (58) or 7 (59). Moreover, reaction severity has also been associated with increasing age (32).

Previous studies have reported an association between atopic diseases and IRs to BLs (60, 61). However, other studies have not found evidences to support that allergologic background such as atopy, elevated specific IgE levels, or rhinitis increase the risk of developing an allergic reaction to BLs (2, 62–64).

A possible existence of some genetic factors involved in allergic reactions BLs has been proposed, as a higher proportion of confirmed allergic reactions has been reported in Europe compared with those in Asia and North America, without significant difference between Asia and North America (65).

In addition, familiar history of drug allergy has been associated in some studies with more prevalence of confirmed BL allergy (31, 59, 63). Indeed, a recent study has created a mathematical model to identify children with low-risk BL allergies where a backward multiple logistic regression showed that a family history of drug allergy was significantly associated with a confirmed BL allergy (66).

Considering issues related to the reaction, the first approach is usually classifying reactions according to the timing of symptom onset following the last dose in IRs and NIRs (2, 47). Several authors have observed a higher proportion of confirmed BL allergy in children reporting IRs compared with those experiencing NIRs (2, 62, 67, 68). However, others could not find any correlation between BL allergy and the timing of reactions (64). Indeed, it has been reported that up to 17% of children reporting NIRs were confirmed as allergic by experiencing an IR in DPT (2, 3, 54, 57, 59, 68–73). This observation suggests limitations in the reliability of reaction chronology registered in clinical history.

Cutaneous manifestations, such as urticaria or maculopapular exanthema, are the most frequently reported, but these symptoms can also be due to viral infections; therefore, differential diagnosis is difficult. However, if these symptoms persist less than 24 h, the child is considered at high risk for being allergic to BL (74, 75). Anaphylaxis is extremely rare in pediatric population, representing less than 0.05% of all cases (32). However, it has been recently identified as moderate to high risk for being allergic to BL, together with the immediate appearance of symptoms (76). Urticaria is the most controversial risk factor for BL allergy because it is included as a high or moderate risk factor in the majority of studies, but some reviews suggest that urticaria appearing more than 1 h after the last dose of BL can be considered as low risk (13, 37, 77). In most pediatric studies, mild cutaneous NIR to BLs is accepted as a low risk for BL allergy, as well as isolated generalized pruritus or gastrointestinal symptoms.

Regarding drugs involved in the reaction, some studies have demonstrated that a higher percentage of allergic patients are confirmed when cephalosporins are implicated (78). This can be explained by the fact that severe bacterial infections are treated with cephalosporins, whereas penicillin or amoxicillin are more frequently associated with viral infections. At this point, it is important to know that consumption patterns may vary between different regions, and that the drugs most commonly implicated are associated with those consumption patterns rather than with a particular drug (79, 80).

Finally, the approach differs between children and adults when the patient is unaware of any information regarding the reaction that resulted in the BL allergy label. While it is considered a moderate or high risk of allergy in the pediatric population, it is considered a low risk in the adult population (74, 81).

## 4. The role of skin testing in delabeling

After clinical history, conventional approach of BL allergy in patients of any age relies on skin testing (42). Sensitivity of STs has been reported to range from 2.6% to 37.8%, while specificity is overall high (96.8%), according to a recent meta-analysis that included 105 primary studies (82–84). However, it is important to take into account that these studies included mainly adults. Determining predictive values for ST with BLs in children is difficult, as only four studies performed DPT regardless of ST results (31, 54, 69, 85). It is of note that these studies assessed children who experienced various adverse reactions related to BLs intake, including not only allergic symptoms but also non-suggestive of allergy ones, such as gastrointestinal symptoms, which limits the generalizability of the results.

The prevalence of positive penicillin STs declines over time, from 27.7% to 0.4% in the period 1980–1993 to 1994–2003 (86). This may be due to changes in prescription patterns in favor of aminopenicillins (87). Therefore, it is important to take into account the determinants used for skin testing. It has been proposed that a standard panel reagents should include major (benzylpenicilloyl octa-L-lysine, BP-OL, DAP®, Diater, and benzylpenicilloyl poly-L-lysine, PPL, Pre-Pen®, AllerQuest LLC) and minor penicillin determinants (sodium benzylpenilloate, DAP®, Diater) (49, 88), amoxicillin, and the culprit BL (89). In this regard, testing clavulanic acid in adults has demonstrated to be beneficial due to its potential for inducing selective reactions (90, 91). Recently, this approach has been extrapolated to the diagnosis workup in pediatric patients (76). However, other authors argue for reducing the panel of STs in children, and propose that testing should only focus on the suspected drug in order to avoid the discomfort associated with STs (92).

The accuracy of STs has been more extensively evaluated in NIRs than in IRs (Table 1). It has been reported that only 3.4%–14% of children with a history of mild NIRs exhibited a positive DPT, experiencing only mild reactions in the DPT (3, 54, 99), which supports the avoidance of ST in non-severe cases (2, 3, 54, 57, 59, 68–73). In addition that STs could be time-consuming and painful, approximately 20% of children have been found to experience fear over their test performance (2). Accordingly, the latest recommendations of many academic societies propose avoiding STs in children particularly in NIRs following a favorable risk assessment (2, 42, 71, 97, 104–107). However, some authors consider that STs are safe and useful, and could avoid exposing children directly to the culprit BLs (58, 62, 92, 95, 99–102, 108) (Table 1). Regarding the utility of STs in NIRs with alarm signs within the spectrum of Severe Cutaneous Adverse Reactions (SCARs), a recent original article has presented findings suggesting that STs may serve as a safe and useful tool for determining the causative drug and assess cross-reactivity (109).

**Table 1:** Studies in which skin tests have been performed, showing the sensitivity of the test and whether the authors are in favor (Pro-ST) or against (Con-ST) performing the test.

*N*	Reagents	Sensitivity (%)	Latency	Pro-ST	Con-ST	Reference
40	PPL/MDM/BP/AX	72	Immediate	Yes	No	(93)
29	PPL/MDM. Culprit	72.4 10.3	Immediate	Yes	No	(94)
1,431	BP/Culprit	86 33.8	Immediate Non-immediate	Yes	No	(62)
50	PPL/MDM/AX	57.1	Both	Yes	No	(95)
52	PPL/MDM/BP/AX	67.31	Immediate	Yes	No	(96)
257	PPL/MDM/BL/AX	69	Both	Yes	No	(97)
290	PPL/MDM/BP/AX	70	Immediate	Yes	No	(98)
229	Culprit	66.6	Both	Yes	No	(92)
105	PPL/MDM/BP/AX/Ampicillin/Cephalosporins	87.5	Non-immediate	Yes	No	(99)
88	PPL/MDM/AX/Culprit	66.7	Non-immediate	No	Yes	(2)
200	AX	14.3	Non-immediate	No	Yes	(69)
352	AX	8	Non-immediate	No	Yes	(70)
732	PPL/MDM/PV/PG/AX/AX-CLV	9.1	Both	No	Yes	(54)
133	PG/AX	0	Both	No	Yes	(59)
1,026	PPL/MDM/BP/Ampicillin/AX/Culprit	75	Non-immediate	Yes	No	(100)
176	PPL/BP/Culprit	13	Both	No	Yes	(71)
126	PG, Ampicillin, AX-CLV, Culprit	54.54	Both	Yes	No	(101)
778	PPL/Pre-Pen/PG/AX/Penicilloate	82.5	Both	Yes	No	(102)
220	PPL/MDM/PG/AX-CLV/Ceftriaxone/Culprit	43.47	Both	No	Yes	(68)
354	Culprit	100	Both	Yes	No	(58)
783	PPL/MDM/AX/AX-CLV/Cefuroxime	28.57 3.8	Immediate Non-immediate	No	Yes	(67)
818	BPO/Pre-PEN	5.9	Immediate	No	Yes	(31)
642	PPL/MDM/AX/PG/Culprit	82.9	Non-immediate	No	Yes	(72)
250	PPL/MDM/AX/Cefuroxime	50	Non-immediate	No	Yes	(3)
158	PPL/BP	20/90	Immediate	No	Yes	(85)
194	Culprit	13.33	Non-Immediate	No	Yes	(57)
213	PPL/MDM/AX/Cefuroxime/PG/AX-CLV	100 10.53	Immediate Non-immediate	No	Yes	(73)

AX, amoxicillin; BP, benzylpenicillin; CLV, clavulanic acid; MDM, minor determinants mixture; PG, penicillin G; PPL, benzylpenicilloyl poly-L-lysine; PV, penicillin V.

## 5. Is there a place for *in vitro* testing?

*In vitro* tests are potential alternative methods that could help reduce the need for risky DPT; however, most of these tests are not clinically validated through well-controlled studies with large series of confirmed patients and controls. Moreover, controversies about their use still exist among American and European Scientific Societies (48). The National Institute of Allergy and Infectious Diseases (NAIAID) from the United States recommends *in vitro* tests for diagnosing IgE-mediated reactions when STs are neither available nor validated (110). By contrast, the European Academy of Allergy and Clinical Immunology (EAACI) has highlighted the major importance of correctly identifying these patients, to avoid severe reactions and also to decrease the percentage of children false-labeled as allergic (111). For that, a recent EAACI position paper (111) and EAACI task force (103) propose the utilization of *in vitro* tests for evaluating immediate severe reactions, as well as mild and severe NIRs.

In general, the decisions concerning *in vitro* testing are common for both adults and children, due to insufficient comparative data and significantly fewer experience and data in children (76). The position paper by the ENDA/EAACI Drug Allergy Interest Group reported good specificity but low sensitivity values in the adult population (112), which limits the diagnostic utility of these tests. The use and choice of *in vitro* tests is based on the mechanism involved, IgE- mediated (immediate) or T-cell-mediated (non-immediate) reactions.

### 5.1. IgE-mediated reactions

#### 5.1.1. Determination of specific IgE

This method is based on the determination of serum drug-sIgE by immunoassay, and the commercial fluoro-enzyme-immunoassay (FEIA) (ImmunoCAP, ThermoFisher, Uppsala, Sweden) is the main *in vitro* procedure in the evaluation of IRs. However, the sensitivity values are limited (0%–50%), and evidences of not optimal specificity have been reported, related to false-positive to penicillin V and the influence of total IgE values (113–117). Moreover, its use is limited because it is only available for some BL structures (benzylpenicilloyl, amoxicilloyl, penicilloyl V, ampicilloyl, cefaclor) (118). Despite these limitations, their performance is recommended prior to *in vivo* tests in severe reactions, as reported by EAACI pediatric task force (106), or in complex cases with negative and/or confusing skin testing as proposed in a recent EAACI position paper (49), in order to reduce the need for DPT.

#### 5.1.2. Basophil activation test

This a functional test based on the analysis of basophil activation in the presence of a stimulus (drug) using flow cytometry. The sensitivity value ranges from 22% to 55%, and the specificity value ranges from 79% to 96% (112, 119). Although it is a non-clinical standardized and validated test, BAT has been reported to be useful as a complementary tool (49), particularly when assessing reactions to BLs that lack a commercially available immunoassay, such as clavulanic acid (51, 120–122) and cefazolin (123, 124). In case of life-threating reactions or high-risk patients, it is recommended to perform BAT prior to *in vivo* tests, including skin testing (49, 112).

### 5.2. Non-IgE-mediated reactions

#### 5.2.1. Lymphocyte transformation test

This is the most commonly used *in vitro* method for detecting T-cell-mediated reactions and is based on the identification of lymphocyte proliferation in the presence of a stimulus (drug). The sensitivity and specificity values range from 58% to 89% and from 85% to 100%, respectively (112). The differences in sensitivity values are related to clinical phenotypes, with higher values in mild and moderate reactions compared with that in severe reactions (112, 118). Although most of the studies refer to adult population, a recent study including 25 children with positive clinical histories of delayed skin reactions to amoxicillin or the amoxicillin–clavulanic acid combination confirmed by DPT showed a lymphocyte transformation test sensitivity of 52% and specificity of 92%, with a positive predictive value of 86% and a negative predictive value of 65% (125). Another study in 17 children with mild NIRs to BLs showed a sensitivity of 52.9% and a specificity of 100% (57). Despite it not being a standardized and validated test and not used routinely, it is recommended in high-risk patients prior to *in vivo* testing (49).

## 6. How to optimize DPT in children?

DPT continues to be widely regarded as the gold standard for confirming or excluding drug allergies (126). Although there is no consensus regarding DPT protocols, there is evidence on the safety and efficacy of DPT in children depending on risk stratification (42, 57, 76, 111, 127–133). One of the first questions that emerge is: when is it considered low, moderate, or high risk? Predictive models and artificial intelligence applications based on historical risk factors have been used to identify variables that can help (134) to elucidate the risk (59, 68, 135–141). In contrast to adults, risk assessment studies in children are scarce, and optimal risk definitions are controversial (76, 103). However, the last practical approaches and algorithms divide patients into low and moderate–high risk. Despite dissimilarities between some articles, there are characteristics that seem to be widely accepted. In some studies, moderate to high-risk patients are considered those who exhibit a reaction in less than 2 h following drug intake, the presence of symptoms compatible with SCARs (such us mucosal lesions, blisters, or desquamation), and/or the presence of one or more of these symptoms (facial swelling, difficulty breathing, lip swelling, wheezing, throat swelling, and drop in blood pressure) (68, 142, 143). On the other hand, low-risk patients are considered those affected with isolated pruritus, delayed urticaria that lasts more than 24 h, palmar exfoliation, or mild maculopapular exanthema.

### 6.1. When can direct DPT be performed?

Direct DPT implies skipping previous skin testing and proceeding to DPT as the unique assessment.

Mild NIRs such as maculopapular eruption and delayed urticaria/angioedema are the most investigated. In a prospective study published in 2021, a single-dose DPT without prior skin testing were performed in 194 children with NIRs, of which 12.4% reacted, but none was severe. Skin tests were conducted exclusively on patients who tested positive for DPT following the confirmation of allergies, showing a 13.33% positivity rate (57). Another study included 153 children who underwent direct DPT, revealing only 1.9% of reactions (128). Both studies concluded that direct DPT is a safe method for mild cutaneous NIRs.

However, little evidence is published for direct DPT for IRs in children. Although it appears to be safe for benign immediate urticaria/angioedema based on several articles, the number of participants included in these studies is limited (128, 130, 144). The largest study to date included a cohort of over 1,900 children with reported history of benign reactions to amoxicillin limited to the skin. The study involved the implementation of direct DPT without prior STs, and the results indicated that only 2.2% of participants experienced mild IRs, while 3.2% experienced NIRs. This study provides further evidence supporting the safe administration of a direct DPT in children with cutaneous symptoms surrounding a treatment with amoxicillin (97).

### 6.2. Using fractionated or single-dose DPT protocols?

The way of performing DPT in children is changing into a simple and less time-consuming manner going for direct and single-dose DPT in selected patients with a favorable risk stratification.

A recent article examined a cohort of 254 children who suffered a NIR to amoxicillin, either alone or in combination with clavulanic, using a graded incremental protocol and prolonged 5-day DPT at home. The study aimed to analyze the duration between the last dose intake and the onset of the observed reaction (129). Interestingly, only 6.5% of children had a positive DPT. Moreover, just one patient had a reaction during the first hour following the first 1/10 dose. The remaining patients experienced reactions more than 2 h after their last dose intake, with the majority of reactions occurring several days later. These results suggest that administering a single-dose DPT may be considered a safe approach for managing mild NIRs in pediatric patients.

In most of the studies where DPT was performed by fractionating the doses, the reactions tended to appear hours or days after the full dose was achieved, when the patient was already at home, which may suggest that a single-dose DPT could potentially offer a comparable level of safety to the multi-step approach, avoiding the need for prolonged hospital stays (69, 130, 131, 144, 145). Based on that, some articles have been published using a single-dose DPT for selected patients with mild NIRs (2, 57, 129, 146, 147). If we compare the percentage of positive DPT in NIRs between fractionated-dose protocols and single-dose protocols, they range from 1.8% to 78.9%, and from 3% to 12.5%, respectively (2, 57, 69, 129–131, 144–147).

## 7. Is a retest needed in children?

In IRs, it is recommended to perform a retest after an initial negative study if the reaction occurred more than 6 months ago due to the potential loss of sensitization over time, in order to avoid potentially severe reactions after subsequent prescriptions of these drugs (148). The rate of repositivization in adults has been reported to be 15%, with a potential increase to 45% in cases of immediate severe reactions (149). The rate of positive retest in children has been reported to be lower, occurring in approximately 2%–5.9% of cases after a positive oral DPT with the culprit (31, 150). The lower rate of resensitization observed in children could be explained by a long-lasting condition, in addition to the possibility that these reactions could be triggered by underlying viral infections, although they are clinically indistinguishable from allergic reactions (2, 151). However, it is important to take into account that in one of these studies, a retest was performed only with PPL and benzylpenicillin but not with amoxicillin, the involved drug in the reaction (31). Despite this, considering the low rate of resensitization, a retest is usually unnecessary in children. Although a retest seems to be unnecessary in mild reactions in children, considering the low rate of resensitization as reported in certain studies, it should be considered in cases of anaphylaxis.

## 8. Novel approaches for delabeling

Delabeling is routinely performed by allergists. However, the number of patients labeled as BL-allergic exceeds the capabilities of examination in many allergy clinics. Taking into account that most patients labeled as BL-allergic can safely receive this antibiotic group, delabeling could be performed by non-allergists in many cases, including pharmacists, nurses, and associate physicians (152). However, the main barrier for delabeling by non-allergists is the lack of training in this area. Therefore, various measures have been developed recently to enhance the management of antibiotic allergy labeling by non-allergists to facilitate their decisions, including educational programs (153), and implementation of visual algorithmic guidelines, digital decision support tools, and electronic health record-incorporated tools and alerts (140, 154–159). These measures have resulted in an increased confidence of non-allergists in managing antibiotic allergy labels and adherence to allergist recommendations, leading to a decrease in the use of broad-spectrum antibiotics for patients who previously reported penicillin allergies (140, 154–159).

Most of these approaches have been carried out in adults, with scarce information being available on the pediatric population. In this regard, delabeling strategies have been designed for implementation in the primary care outpatient setting, as this is the best way to reach the largest number of children with BL allergy label. It has been proposed to conduct an initial telemedicine consultation in order to screen children for a low probability of true penicillin allergy (replacing the outpatient visit), followed by a single-dose oral DPT in an outpatient setting (146). This approach was cost-saving during the COVID-19 pandemic (160), but more studies are required to consider whether this model, in primary care or as an entirely nurse-led procedure, will continue to be of value in the aftermath of Covid-19. The Standards of Care Committee of the British Society for Allergy and Clinical Immunology (BSACI) have developed a guideline to identify patients at low risk of penicillin true allergy and a framework for conducting DPT by non-allergists (161). Recently, a delabeling protocol has been developed by pediatricians to identify low-risk patients who may be allergic to BL, showing to be safe and viable for being implemented in a pediatric primary care clinic (162).

## 9. Conclusions

Most BL allergy labels are acquired during childhood, but only a small proportion of these patients have a true allergic response. However, this is rarely verified, and the label is carried out over adulthood, with a negative impact as they have been associated with less effective therapies, emergence of multidrug-resistant organisms, and prolonged hospital stays. Therefore, an allergological evaluation is crucial to address the delabeling of these patients, and to initiate this evaluation early in childhood to avoid false labeling in adult life. However, despite the important consequences and the high prevalence of the label of BL allergy, its management remains controversial. Due to the scarcity of studies conducted in children, the current recommendations are the same as those established for adults. The prevalence of positivity in STs among children is low, and the role of *in vitro* testing in this population is not well defined, being DPT considered the gold standard to confirm or discard the diagnosis of allergy and typically conducted only when preliminary tests are negative. However, novel strategies have been implemented in order to optimize a protocol for BL allergy diagnosis in the pediatric population. Several studies have demonstrated that it is possible to identify children who are at low risk of a true BL allergy and performing DPT without conducting prior skin testing in those showing non-severe reactions.

There have been significant efforts to expand BL allergy evaluations beyond allergists in order to reach the largest number of children with BL allergy label. However, more research is needed before delabeling by non-allergists can become a standard of care.
