# Corrigendum: Approach for delabeling beta-lactam allergy in children

**DOI:** 10.3389/falgy.2023.1361973

**Published:** 2024-01-12

**Authors:** R. Sáenz de Santa María, G. Bogas, M. Labella, A. Ariza, M. Salas, I. Doña, M. J. Torres

**Affiliations:** ^1^ Allergy Unit, Hospital Regional Universitario de Málaga, Hospital Civil, Málaga, Spain; ^2^ Allergy Research Group, Instituto de Investigación Biomédica de Málaga-IBIMA, Hospital Civil, Málaga, Spain; ^3^ Nanostructures for Diagnosing and Treatment of Allergic Diseases Laboratory, Andalusian Center for Nanomedicine and Biotechnology-BIONAND, Parque Tecnológico de Andalucía, Málaga, Spain; ^4^ Departamento de Medicina, Universidad de Málaga, Facultad de Medicina, Málaga, Spain

**Keywords:** anaphylaxis, beta-lactam, children, drug provocation test, maculopapular exanthema, skin test, urticaria

A Corrigendum on Approach for delabeling beta-lactam allergy in children By Sáenz de Santa María R, Bogas G, Labella M, Ariza A, Salas M, Doña I, Torres MJ. (2023). Front Allergy. 4:1298335. doi: 10.3389/falgy.2023.1298335

In the published article, there was an error in the Funding statement. The following was incorrectly written: ‘The present study has been supported by Institute of Health “Carlos III” of the Ministry of Economy and Competitiveness (grants cofunded by European Regional Development Fund (ERDF): PI21/00329, and Redes de Investigación Cooperativa Orientadas al Resultado en Salud: Enferemedades Inflamatorias (RD21/0002/0008).’

This has been replaced by ‘The present study has been funded by Instituto de Salud Carlos III through the project “PI21/00329” and co-funded by the European Union.’

The full, correct Funding statement appears below.

